# Multiplex Determination of K-Antigen and Colanic Acid Capsule Variants of *Cronobacter sakazakii*

**DOI:** 10.3390/genes15101282

**Published:** 2024-09-29

**Authors:** Khaled M. Ibrahim, Abdlrhman M. Alsonosi, Mahmoud B. Agena, Bassam A. Elgamoudi, Stephen J. Forsythe

**Affiliations:** 1 Microbiology Department, Libyan Biotechnology Research Center, Tripoli P.O. Box 30313, Libya; 2 Microbiology Department, Faculty of Medicine, Sebah University, Sebha P.O. Box 1000, Libya; 3 Libyan Medical Research Center, Zawia P.O. Box 20311, Libya; 4 Institute for Biomedicine and Glycomics, Griffith University, Southport, QLD 4215, Australia; 5 Foodmicrobe.com Ltd., Nottingham NG12 5GY, UK

**Keywords:** *C. sakazakii*, capsule variants, K-antigen and colanic acid

## Abstract

*Cronobacter sakazakii* is associated with the ingestion of contaminated reconstituted powdered infant formula (PIF), resulting in necrotizing enterocolitis, sepsis and meningitis in neonatal infants. Potential virulence determinants include the variable capsular polysaccharides; K-antigen and colanic acid (CA). Strains encoding for the capsule variant K2:CA2 have been strongly associated with neonatal meningitis cases. This study aimed to develop and apply a multiplex PCR assay to determine *C. sakazakii* K-antigen and colanic acid types. Twenty-six strains of *C. sakazakii* which had previously been isolated from food and environmental sources were used. These cover 18 multilocus sequence types and four serotypes. Based on our research findings, we have identified two K-antigen types present. Specifically, the K1-antigen was observed in sequence types ST1, ST8, ST20, ST23, ST64, ST198, ST263, ST264 and ST406, while the K2-antigen was present in ST4, ST9, ST12, ST13, ST136, ST233, ST245 and ST405. Additionally, we detected colanic acid (CA) type 1 in sequence types ST1, ST8, ST9, ST20, ST245 and ST405, and colanic acid (CA) type 2 in ST4, ST12, ST13, ST23, and ST64. We compared the predicted K-antigen and colanic acid types with the entire genome sequences of the strains. The comparison showed complete agreement between the PCR amplification results and the genomic analysis of the K-antigen and colanic acid-encoding regions. This assay is a useful tool for rapid identification of *C. sakazakii*, K-antigen and colanic acid types, in routine diagnoses and foodborne investigations. In addition, it will contribute to our knowledge of virulence factors associated with life-threatening neonatal meningitis.

## 1. Introduction

*Cronobacter* spp. (former *Enterobacter sakazakii*) are opportunistic bacterial pathogens which can be isolated from a wide range of foods and environmental sources [1,2]. Serious infections of *Cronobacter* are associated with neonates, particularly those with low birth weight (<1.5 kg) and <28 days in age. Such infections may result in necrotizing enterocolitis (NEC), septicemia, and meningitis with high fatality rates (40–80%) [3,4,5]. Bowen and Braden (2006) [6] reported that surviving neonatal cases of *Cronobacter* meningitis may have severe neurological damage. Infections also occur in adults, in particular, immunocompromised patients [7,8,9]. Adult infections are associated with bacteremia, wound infections and urosepsis. *C. sakazakii* and *C. malonaticus* are the species isolated from the majority of clinical samples in both age populations (neonatal and Adult) [2,5,10].

Several studies have reported that *Cronobacter* species isolated from different environments such as powdered infant formula (PIF) and milk powder production factories including floors, roofs, tanker bays, drying towers, roller dryers, conveyors, and air filters of industrial units [11,12,13,14]. *Cronobacter* species can persist in these environments because of their ability to survive spray drying, desiccation and osmotic stress [15]. Caubilla-Barron and Forsythe (2007) [16] reported that *Cronobacter* species can persist and survive more than 2 years in PIF. The ingestion of contaminated PIF is the main route of infant infection, and this has led to the development of internationally approved detection methods for the food industry [2].

Capsular polysaccharides of Gram-negative bacteria play a significant role in maintaining the structural integrity of the cell in hostile environmental conditions. The polysaccharide capsules are major bacterial virulence factors and environmental fitness traits [17]. Due to its propensity for water, the capsule will contribute to the organisms’ persistence under desiccated conditions in natural and food production environments [2,18].

The O-antigen and K-antigen in Gram-negative bacteria consist of long polysaccharide units, which are covalently linked to lipid A in the outer membrane. This diversity has been the basis for differentiation methods of *E. coli* and *Salmonella* [19]. *E. coli* produces more than 80 different capsular polysaccharide K-antigens, while there are over 2500 different *Salmonella* serotypes [20]. Consequently, polysaccharide capsule diversity can be used as a taxonomic tool within the Enterobacteriales [21].

The K-antigen gene cluster of *E. coli* consists of three genomic regions. Region 1 includes the *kpsEDCS* genes and Region 3 includes *kpsTM* encode for enzymes and transport proteins responsible for the initiation of chain elongation and translocation to the cell surface. Variable Region 2 genes encode the glycosyltransferases and other enzymes responsible for the biosynthesis of the K-antigen [17].

Kaczmarek et al. (2014) [22] reported that the K1 antigen is a key virulence determinant of *E. coli* strains and has been associated with meningitis, bacteremia and septicemia, particularly in neonatal cases. Neonatal meningitis *Escherichia coli* (NMEC) is a predominant Gram-negative bacterial pathogen associated with meningitis in babies. NMEC is also associated with strains possessing capsular polysaccharides [23].

A multiplex PCR assay targeting a capsular polysaccharide synthesis gene cluster of *Klebsiella* serotypes K1, K2 and K5 was evaluated using reference serotype strains and a panel of clinical isolates. The PCR assay was highly specific for serotypes associated with virulence in humans [24]. Feizabadi et al. (2013) [25] proposed a rapid and reliable PCR method for the identification of *K. pneumoniae* K1 and K2 serotypes.

A genomic study of 11 *Cronobacter* strains by Joseph et al. (2012) [26] notes that the *Cronobacter* possessed a sequence-type variable capsular polysaccharide encoding region. Later studies by Ogrodzki and Forsythe (2015 and 2017) [27,28] reported that it is homologous with the K-antigen of *E. coli* and is found in all *Cronobacter* species. The *Cronobacter* K-antigen is encoded in three regions, of which most of Region 1 *(kpsEDCS)* and all Region 3 *(kpsTM)* were conserved across the *Cronobacter* genus. The glycosyltransferase genes in Region 2 varied in length and CG % content. Additionally, the terminal sequence of kspS (Region 1) differed in conjunction with the variation in Region 2. Since Region 2 encodes for glycosyltransferases, the two K-antigens (K1 and K2) lead to the production of two distinct exported polysaccharides. Furthermore, a comparison of kpsS (which encodes for the capsular polysaccharide transport protein) revealed sequence variation in accordance with kps Region 2 [27]. The chemical composition of Cronobacter K-antigen is still unknown; however, K2 is linked to strains from neonatal meningitis cases.

Another bacterial capsular polysaccharide is known as colanic acid (CA). This is associated with bacterial protection against desiccation, extreme temperatures and acidic environmental conditions [29,30]. In *Cronobacter* species, the colanic acid-encoding gene cluster is located adjacent to the O-antigen region and separated by the *galF* gene [27]. Furthermore, Ogrodzki and Forsythe (2015) [27] reported that there are two variants in the colanic acid-encoding gene cluster. CA1 is composed of 21 genes, while CA2 lacks the *galE* gene (encoding for UDP-N-acetyl glucosamine 4-epimerase). *C. sakazakii* and *C. malonaticus* isolates with capsular type [K2:CA2:Cell+] were associated with neonatal meningitis and necrotizing enterocolitis. Other capsular types were less associated with clinical infections [27].

This study aimed to develop and apply a multiplex PCR assay targeting the *Cronobacter* capsular polysaccharide genes *kpsS* (K1 and K2) and *galE* (CA1 and CA2). This assay could subsequently be useful for the specific detection, and rapid and simple identification of K-antigen and colanic acid types, respectively.

## 2. Materials and Methods

### 2.1. Bacterial Strains

Twenty-six strains of *C. sakazakii* were used in this study. These strains were from the culture collection of *Cronobacter* spp. of Nottingham Trent University (NTU). These strains had previously been isolated from various food and environmental sources. They were from 18 different sequence types and 4 serotypes O:1, O:2, O:3 and O:4; Table S1.

### 2.2. Genomic DNA Extraction

Genomic DNA was prepared according to the instructions of the manufacturer, 1.5 mL of culture grown overnight in TSB using the GenElute™ Bacterial Genomic DNA Kit (Sigma-Aldrich, London, UK). The purity and concentration of the extracted DNA were measured by using Nanodrop 2000 (Thermo Scientific, London, UK).

#### 2.2.1. *C. sakazakii* K-Antigen Profiling

The K-capsule encoding region in *Cronobacter* spp. is composed of three regions. The genomic study indicated that the variations between K1 and K2 capsule types were attributed to Region 2 and within the *kpsS gene* (encoding for the capsular polysaccharide transport protein) of Region 1 [27]. Therefore, K1 and K2 primers were designed based on the capsular gene (*kpsS*) and were identified from the sequence information of *C. sakazakii* strain 658 (*kpsS1*) and strain 6 (*kpsS2*), respectively. Primers flanking K1 and K2 were designed using the Primer 3.0 software and were synthesized by Eurofins MWG Operon (London, UK). The primer names, their sequence and predicted product size are summarized in (Table S2).

#### 2.2.2. K-Antigen Gene Amplification

The multiplex PCR was performed by mixing two primers (K1 and K2) in a final volume of 50 µL containing the following components: 1× dreamtaq buffer; 2.5 mM MgCl2; 400 µM concentrations (each) of dATP, dCTP, dGTP, and dTTP; 0.06 to 0.10 µM primer; and 1 U of DreamTaq DNA polymerase and DNA template (200 ng). The following PCR conditions were used for amplification: an initial denaturation step at 95 °C for 5 min, followed by 30 cycles of 94° C for 30 s, 59 °C for 30 s, and 72 °C for 1 min, with a final extension at 72 °C for 8 min. Five microliters of the PCR products were loaded in 1.5% agarose gel in 1× TAE buffer) at a voltage of 70 V for ~60 min. 

#### 2.2.3. *C. sakazakii* Colanic Acid Profiling

Colanic acid variant primers were based on the *galE* gene sequence encoding for UDP-N-acetyl glucosamine 4-epimerase of *C. sakazakii* strain 658. They were designed using the Primer 3.0 software, and obtained from Eurofins MWG Operon company (London, UK); Table S3.

#### 2.2.4. *C. sakazakii* Colanic Acid Amplification

The PCR conditions used for amplification were an initial denaturation step at 95 °C for 5 min, followed by 30 cycles of 94 °C for 30 s, 60 °C for 30 s, and 72 °C for 1 min, with a final extension at 72 °C for 8 min. Five microliters of the PCR products were loaded in 1.5% agarose gel in 1X TAE buffer) at a voltage of 75 V for ~60 min.

Genomic investigation, In silico analyses were carried out using the accessible *Cronobacter* genomes available by open access at the PubMLST *Cronobacter* database (www.pubmlst.org/cronobacter/) (accessed on 29 January 2018). The *Cronobacter* genomes included in this study were *C. sakazakii* strains; 1844, 1882, 1992, 1886, 1888, 1906, 1105, 377, 658, 1885, 2027, 1890, 1847, 1845, 1881, 1887, 1283, 1889, 1108 and 1908.

## 3. Results

### 3.1. Sequence Type (ST) and Serotype Determination

Twenty-six strains of *C. sakazakii* were used in this study. These strains were from the culture collection of *Cronobacter* spp. of Nottingham Trent University (NTU). The results of the Sequence type (ST) and O-antigen serotyping for 26 strains are included in Table S1. In this study, strains were divided into four O-antigen serotypes O:1, O:2, O:3 and O:4. serotype O:2 was the most dominant serotype in studied *C. sakazakii* strains, which was confirmed to be particularly predominant in clinical cases [31].

#### 3.1.1. K-Antigen 

**Figure 1:**
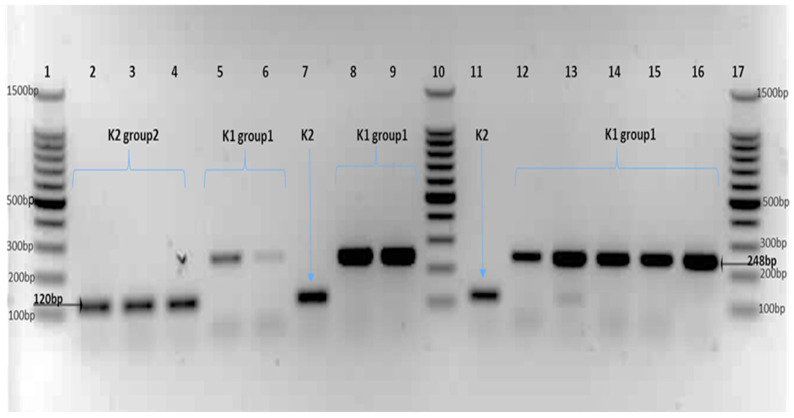
PCR amplification of K1 and K2-antigen type in *C. sakazakii* strains. Lane 1: 100 bp DNA Ladder marker. Lane 2: strain 1908. Lane 3: strain 1107. Lane 4: strain 1564. Lane 5: strain 1906. Lane 6: strain 1888. Lane 7: strain 1105. Lane 8: strain 1283. Lane 9: strain 1884. Lane 10: 100 bp DNA Ladder marker. Lane 11: strain 377. Lane 12: strain 1990. Lane 13: strain 1884. Lane 14: strain 1885. Lane 15: strain 1843. Lane 16: strain 1882. Lane 17: 100 bp DNA Ladder marker.

PCR amplification of K1 and K2 from *C. sakazakii* strains is shown in Figure 1. The predicted PCR amplicon sizes are 248 bp and 120 bp for K1 and K2, respectively (Figure 1). The K1 capsular type was noted in *C. sakazakii* strains with STS; ST1, ST8, ST20, ST23, ST64, ST198, ST263, ST264 and ST406. Whereas K2 was primarily found in *C. sakazakii* sequence types ST4, ST9, ST12, ST13, ST136, ST233, ST245 and ST405. The PCR product size of K-antigen type (K1 and K2) was compared with the genome investigation of studied strains as shown in Table S2. The PCR product size of K-antigen type (K1 and K2) was compared with the genome investigation of studied strains (Region 2) as shown in Table S1. The comparison showed an agreement between PCR amplification results and the genomic study of K-antigen Region 2.

#### 3.1.2. Colanic Acid (CA) 

**Figure 2:**
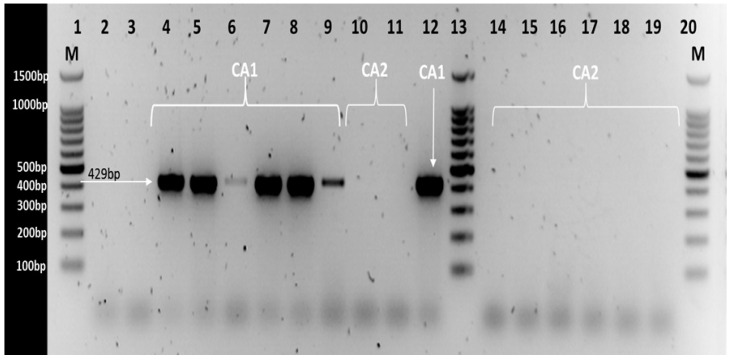
PCR amplification of *galE* gene in *C. sakazakki* strains. Lane 1: 100 bp DNA Ladder Marker. Lane 2: the negative control (with no bacterial DNA). Lane 3: strain 377 as a positive control for CA2 (no PCR product). Lane 4: strain 1107. Lane 5: strain 1283. Lane 6: strain 1888. Lane 7: strain 1906. Lane 8: strain 1844. Lane 9: strain 1882. Lane 10: strain 1105. Lane 11: strain 1564. Lane 12: strain 1 as a positive control for CA1. Lane 13: 100 bp DNA Ladder Marker. Lane 14: strain 1843. Lane 15: strain 1884. Lane 16: strain 1907. Lane 17: strain 1908. Lane 18: strain 1990. Lane 19: strain 1886. Lane 20: 100 bp DNA Ladder Marker.

PCR amplification of CA1 for *C. sakazakii* strains was shown in Figure 2. Colanic acid type 1 (CA1) was found in the majority of *C. sakazakii* sequence types; ST1, ST8, ST9, ST20, ST245 and ST405 with PCR product size 429 bp. At the same time, CA2 was found in *C. sakazakii* sequence types ST4, ST12, ST13, ST23 and ST64. The later strains showed no PCR products due to the absence of the *galE* gene (Figure 2). Table S1 shows the agreement between the PCR determination and genome investigation for the CA type of studied strains.

### 3.2. Comparison between PCR Amplification Result and the Genomic Investigation

The K-antigen type 1 (K1) strains produced PCR bands of the expected size of 248 bp, whereas K-antigen type 2 (K2) strains produced PCR bands of the expected size 120 bp (Figure 1). The PCR product size of K-antigen type (K1 and K2) was compared with the genome investigation of studied strains; see Table S1. The genomic information of these strains was obtained from accessible open access at the PubMLST *Cronobacter* database (www.pubmlst.org/cronobacter/) (accessed on 29 January 2018). The comparison showed complete agreement between the PCR amplification result and the genomic study of K-antigen Region 2.

According to the genome investigation, two variants were found within the colanic acid (CA) cluster, CA1 and CA2. These were composed of 21 and 20 genes, respectively, which differed in the presence of *galE* in CA1 (21 genes), and absence in CA2 (20 genes). Therefore, primers were designed based on *galE* gene sequence (presence of *galE,* CA1).

The colanic acid type 1 (CA1) strains produced a PCR product size of 429 bp, while CA2 strains showed no PCR products as a result of the absence of the *galE* gene (Figure 2). Table S1 showed also an agreement between the PCR determination and genome investigation for the CA type of studied strains (presence/absence of *galE* gene).

## 4. Discussion

Studies by Ogrodzki and Forsythe (2015 and 2017) [27,28] indicate that it is homologous to the K-antigen of E. coli and is present in all Cronobacter species. As previously reported, most of the K-antigen Region 1 (*kpsEDCS*) and all Region 3 (*kpsTM*) in *Cronobacter* species were conserved across the genus; however, there are two variants in Region 2 which are diverse in their CG % content and length [27]. The K-antigen-specific CPS composition is currently unknown; however, it is of interest as it may be an important virulence or environmental fitness trait. Moreover, the K-antigen Region 1 (*kpsEDCS*) and Region 3 (*kpsMT*) genes were found in all *Cronobacter* spp., and the highly variable Region 2 genes were assigned to two homology groups, K1 and K2 types. These variations between K1 and K2 capsule types were attributed to the *kpsS* gene of Region 1, and the entire Region 2 (3 genes).

Similarly, there are two variants in the colonic acid synthesis gene cluster which are located adjacent to the O-antigen region and separated by the *galF* gene: differing in the presence/absence of *galE* [27]. *C. sakazakii* isolates with capsular type [K2:CA2] are associated with neonatal meningitis and necrotizing enterocolitis, and other capsular types are less associated with clinical infections [27,28].

The purpose of this study was to develop and validate a multiplex PCR assay targeting capsular polysaccharide genes *kpsS* (K1 and K2) and *galE* (encoding for UDP-N-acetyl glucosamine 4-epimerase), CA1 and CA2 for the specific detection, rapid and simple identification of K-antigen and colanic acid type, respectively. Twenty-six *C. sakazakii* strains were used in this study, these strains were isolated from food and environmental sources, covering different STs (*n* = 18). Initial PCR-serotyping assays revealed that they were in four serotypes (O:1,O:2,O:3 and O:4) (Table S1).

Colanic acid type 1 (CA1) were found in *C. sakazakii* sequence types such as ST1, ST8, ST9, ST20, ST245 and ST405, while CA2 was primarily found in *C. sakazakii* sequence types ST4, ST12, ST13, ST23, ST42, ST64, ST136, ST198, ST233, ST263, ST264 and ST406; Table S1. These are clinically important sequence types concerning neonatal infections sequence types in particular ST4 and ST12. No cross-reactions were observed between the specific two primers of capsular type K1 and K2 (Figure 2). Moreover, Table S1 shows the agreement between the PCR determination and genome investigation for the CA type of studied strains.

Until recently, there were only 18 *Cronobacter* serotypes across the whole genus, with only 7 in *C. sakazakii* [31]. In this study, strains were divided into four O-antigen serotypes, O:1, O:2, O:3 and O:4. Serotype O:2 was the most dominant serotype in studied *C. sakazakii* strains, which was confirmed to be particularly predominant in clinical cases [31]. The same serotype occurs in different ST. For example, ST4 strains had three serotypes, which are O:2, O:3 and O:4. Furthermore, there is no clear correlation between serotype and K-antigen or colanic acid type. For example, the O:2 serotype covers three different capsule profiles, O:2:K1:CA1, O:2:K2:CA2 and O:2:K2:CA1.

The most dominant capsule profile was K2:CA2, this includes strains with ST4, ST12 and ST13. These sequence types are strongly associated with severe neonatal infections of meningitis and NEC [32]. However, strains belonging to other STs may also cause severe neonatal infections such as bacteremia and septicemia. The capsule profile, sequence type ST and serotype analyses suggested that *Cronobacter* strains isolated from food and environmental sources were highly diverse. This was particularly notable for the isolates, which were obtained from different foods.

## 5. Conclusions

PCR assays targeting the capsular polysaccharide genes, such as the K-antigen, are useful for pathogenicity and taxonomic studies. However, genomic prediction of K-antigen (K1 and K2) and colanic acid type (CA type) may not be feasible for laboratories with limited budgets. Instead, targeted methods based on PCR amplification may be more accessible to determine the K-antigen and colanic acid type in *C. sakazakii*. Thus, this multiplex assay may help in *C. sakazakii* capsular type identification in routine diagnoses.

## Data Availability

This study used the *Cronobacer* PubMLST open access database, as described by Jolley et al. (2018) [33]. Wellcome Open Res 3:124., all relevant data, sequence type (ST), serotype and whole genome sequence are available via *Cronobacer* PubMLST open access database https://pubmlst.org/bigsdb?db=pubmlst_cronobacter_isolates, accessed on 29 January 2018.

## Supplementary Materials

The following supporting information can be downloaded at: https://www.mdpi.com/article/10.3390/genes15101282/s1, Table S1: K-antigen type, colanic acid type (CA) and serotype profiles of *C. sakazakii* strains according to PCR determination and genome investigation; Table S2: K-antigen type primer name, target gene, primer sequence and product size; Table S3: CA-type primer name, target gene, primer sequence and product size.
