# Supplementary material for: Multiplex Determination of K-Antigen and Colanic Acid Capsule Variants of Cronobacter sakazakii
Source: Genes (Basel). 2024 Sep 29;15(10):1282. doi: 10.3390/genes15101282 (PMC11507822; doi:10.3390/genes15101282)
Supplement: Supplementary file 1 [file genes-15-01282-s001.zip › genes-3112490-supplementary.pdf]

Article

Multiplex determination of K-Antigen and Colanic Acid Capsule Variants of *Cronobacter sakazakii*

Khaled M Ibrahim [1,*], Abdlrhman M Alsonosi [2], Mahmoud B Agena [3], Bassam A Elgamoudi [4,*] and Stephen J Forsythe [5]

Table S1. K-antigen type, colanic acid type (CA) and serotype profiles of *C. sakazakii* strains according to PCR determination and genome investigation. *ST: sequence type, *WGS: whole genome sequence, *NG: no genome sequence.

Strain number	Source	ST	O-antigen	K-antigen PCR assay	K-antigen WGS	Colanic acid (CA) PCR assay	Colanic acid (CA) WGS
658	Infant formula	1	O:1	K1	K1	CA1	CA1
1906	Environmental	8	O:1	K1	K1	CA1	CA1
1888	Food	8	O:1	K1	K1	CA1	CA1
1843	Spice	23	O:2	K1	NG	CA2	NG
1890	Food	42	O:2	K1	K1	CA2	CA2
1881	Food	64	O:2	K1	K1	CA2	CA2
1884	Herb	263	O:2	K1	NG	CA2	NG
1990	Food	264	O:2	K1	NG	CA2	NG
1885	Herb	406	O:2	K1	K1	CA2	CA2
2027	Food	406	O:2	K1	K1	CA2	CA2
1283	Food	8	O:3	K1	K1	CA1	CA1
1882	Food	20	O:3	K1	K1	CA1	CA1
1889	Food	198	O:4	K1	K1	CA2	CA2
1847	Milk powder	245	O:1	K2	K2	CA1	CA1
1105	Milk powder	4	O:2	K2	K2	CA2	CA2
377	Weaning food	4	O:2	K2	K2	CA2	CA2

1107	Weaning food	9	O:2	K2	NG	CA1	NG
1887	Food	13	O:2	K2	K2	CA2	CA2
1992	Food	136	O:2	K2	K2	CA2	CA2
1845	Food	233	O:2	K2	K2	CA2	CA2
1564	Food	4	O:3	K2	NG	CA2	NG
1886	spice	4	O:3	K2	K2	CA2	CA2
1844	Food	405	O:3	K2	K2	CA1	CA1
1907	Environmental	4	O:4	K2	NG	CA2	NG
1908	Environmental	4	O:4	K2	K2	CA2	CA2
1108	Weaning food	12	O:4	K2	K2	CA2	CA2

Table S2. K-antigen type primer name, target gene, primer sequence and product size

K-type	Target gene	primer sequence	Tm	Product Size (bp)	Reference
K1	*kpsS1*	F- CGTATCACACCCTCGCTACT<br>R- TTAAACACCTGAAGCACCGC	59	248	This study
K2	*kpsS2*	F- TTATATGCAGTTGCCCGACA<br>R-TATGAACGATGATACGCCCA	59	120	This study

Table S3. CA-type primer name, target gene, primer sequence and product size.

CA-type	Target gene	primer sequence	T_m	Product Size (bp)	Reference
CA1	*galE*	F- GTCGGCACCAGACTGATTGA<br>R-TAAAGAACGGGTATCCGGCG	60	429	This study
